# Predicting ovarian function loss after chemotherapy and anti-HER2 therapy in young breast cancer patients

**DOI:** 10.1093/jnci/djaf198

**Published:** 2025-08-12

**Authors:** Matteo Lambertini, Deirdre Allegranza, Ruediger P Laubender, Nadia Harbeck, Sandra M Swain, Charles E Geyer, Dennis J Slamon, Gabriella Bobba, Chiara Lambertini, Sanne de Haas, Eleonora Restuccia, Ines Vaz-Luis, David A Cameron, Ian E Krop, Eric P Winer, Richard A Anderson

**Affiliations:** Department of Internal Medicine and Medical Specialties (DIMI), School of Medicine, University of Genova, Genoa, Italy; Department of Medical Oncology, U.O. Clinica di Oncologia Medica, IRCCS Ospedale Policlinico San Martino, Genoa, Italy; Clinical Development and Medical Affairs, Roche Diagnostics International Ltd, Rotkreuz, Switzerland; Biostatistics & Data Science Core Lab & Near Patient Care, Roche Diagnostics GmbH, Penzberg, Germany; Breast Center, Department of OB&GYN and CCC Munich, LMU University Hospital, Munich, Germany; Georgetown University Medical Center, Lombardi Comprehensive Cancer Center, Washington, DC, United States; MedStar Health, Washington, DC, United Statesz; Department of Medicine, UPMC Hillman Cancer Center, Pittsburgh, PA, United States; David Geffen School of Medicine at UCLA, Los Angeles, CA, United States; Clinical Development and Medical Affairs, Roche Diagnostics International Ltd, Rotkreuz, Switzerland; Translational Medicine Oncology, F. Hoffmann-La Roche Ltd, Basel, Switzerland; Translational Medicine Oncology, F. Hoffmann-La Roche Ltd, Basel, Switzerland; Product Development Oncology, F. Hoffmann-La Roche Ltd, Basel, Switzerland; Cancer Survivorship Group, Inserm Unit 981, Gustave Roussy, Villejuif, France; Department for the Organization of Patient Pathways, Gustave Roussy, Villejuif, France; Institute of Genetics and Cancer, Western General Hospital—NHS Lothian, Edinburgh, United Kingdom; Yale Cancer Center, Yale School of Medicine, New Haven, CT, United States; Yale Cancer Center, Yale School of Medicine, New Haven, CT, United States; Centre for Reproductive Health, Institute for Regeneration and Repair, University of Edinburgh, Edinburgh, United Kingdom

## Abstract

**Background:**

The ability to predict ovarian function loss after anticancer treatment is important for appropriate oncofertility counseling and to aid in therapy decision-making for young women with early breast cancer (eBC).

**Methods:**

This biomarker analysis of the BETH (NCT00625898) and KAITLIN (NCT01966471) randomized trials investigated anti-Müllerian hormone (AMH) use, alone and combined with follicle stimulating hormone (FSH) and estradiol (E2), for predicting ovarian function loss following currently adopted chemotherapy and anti-HER2 therapy in premenopausal women with HER2-positive eBC. Serum samples were centrally tested measuring AMH, FSH, and E2 using Roche Elecsys assays.

**Results:**

Among 194 included patients (BETH: *n* = 62; KAITLIN: *n* = 132), AMH values declined from baseline median 8.44 pmol L^−1^ to undetectable levels (<0.07 pmol L^−1^) at the end of therapy, with partial recovery at 36 months (median 0.14 pmol L^−1^). AMH measured at baseline was predictive of ovarian loss (area under the ROC curve [AUC] = 0.784). Addition of age to AMH slightly improved AUC to 0.800. AMH measured at the end of therapy had AUC 0.741, which increased to 0.785 with addition of age. The combination of AMH at baseline and end of therapy increased prediction to 0.808 and with addition of age to 0.820. Addition of baseline FSH and E2 did not improve prediction in any analysis.

**Conclusions:**

These results support the use of pretreatment measurement of AMH in predicting ovarian function loss in premenopausal women with HER2-positive eBC receiving chemotherapy and anti-HER2 therapy. Measurement of AMH at the end of treatment had reduced accuracy than pretreatment but in combination added slightly to the value of pretreatment sampling.

## Introduction

In premenopausal women, breast cancer is the most commonly diagnosed malignancy as well as the leading cause of mortality.^1^ Additional age-related issues should be considered in the management of breast cancer in premenopausal patients; prominent among them is the risk of developing treatment-induced premature ovarian insufficiency (POI) in light of their increasingly high chances of being cured and long survivorship nowadays.^2^ Infertility and early menopause with consequent wide-ranging adverse health effects occurring with POI are of great concern to many women.^3^^,^^4^ The risk of gonadotoxicity with the proposed systemic anticancer therapies is a core component of oncofertility counseling for all patients diagnosed during their reproductive years.^5^^,^^6^ Being able to estimate the risk of developing treatment-induced POI is essential for counseling and guiding decisions on available strategies for fertility and/or ovarian function preservation.

The status of ovarian function following cytotoxic therapy is also crucial for optimal endocrine therapy choices in premenopausal women, particularly regarding the need for adjuvant ovarian function suppression.^7^ Most of the available evidence to estimate the risk of treatment-induced gonadotoxicity in patients with breast cancer is from cyclophosphamide- and anthracycline-based regimens, with limited or no data with the use of taxane-based regimens and targeted therapies.^5^^,^^6^ In addition, most of the available data on treatment-induced gonadotoxicity are based on the presence or absence of amenorrhea a few months to years following completion of therapy. However, substantial gonadotoxicity from systemic anticancer therapies may occur without loss of menses,^8^^,^^9^ and ovarian function recovery can occur even beyond 1 or 2 years following chemotherapy.^10-12^ Hence, more accurate ways to estimate the risk of ovarian function loss following systemic anticancer therapies are needed.

Prediction of future ovarian activity requires a biomarker that reflects long-term function of the ovary, distinct from the short-term changes that reflect cyclic ovulation. Anti-Müllerian hormone (AMH) is produced by the small growing follicles of the ovary, and not by the nongrowing primordial follicles nor the later preovulatory stages. This results in intracycle stability, unlike with follicle stimulating hormone (FSH) and estradiol (E2), which increases clinical utility.^13^ However, it is partially sensitive to suppression by hormonal contraception.^14^ Measurement of AMH has become established in assisted reproduction as a reliable predictor of the ovarian response to stimulation,^15^^,^^16^ and there is now substantial evidence that AMH has value in the context of natural aging,^17^^,^^18^ as well as in women with cancer.^19^ In addition to a progressive decline with age from a peak in the mid-20s following a rise through childhood and adolescence,^20^ AMH concentrations fall rapidly and markedly during treatment with cytotoxic chemotherapy.^19^ Initial studies in women with breast cancer suggested that pretreatment AMH levels were predictive of subsequent, postrecovery, ongoing ovarian activity as reflected by continuing menses.^21^^,^^22^ Subsequent studies have confirmed these findings,^23^^,^^24^ and have also suggested that measurement shortly after completion of chemotherapy may have predictive value for later ovarian function in women more than 40 years of age at breast cancer diagnosis.^25^^,^^26^

These findings require confirmation in different settings and over wider age ranges, using current chemotherapy regimens and other targeted treatments. Given the clinical utility in fertility preservation counseling and for consideration of subsequent endocrine treatment approaches, the present analysis aimed at investigating the potential for AMH and other ovarian biomarkers to assess posttreatment long-term loss of ovarian function and its pretreatment prediction in premenopausal women with HER2-positive early breast cancer who participated in 2 large randomized controlled trials (RCTs) employing modern chemotherapy regimens and HER2-directed therapies.

## Methods

### Study design and participants

BETH (NCT00625898) and KAITLIN (NCT01966471) were RCTs investigating adjuvant chemotherapy plus anti-HER2 therapy in patients with high-risk HER2-positive early breast cancer.^27^^,^^28^ For the purpose of this analysis, only samples from patients who received currently adopted treatments were used, ie, cohort 1A (6 cycles of docetaxel/carboplatin plus trastuzumab) and cohort 2A (3 cycles of docetaxel plus trastuzumab followed by 3 cycles of anthracycline- and cyclophosphamide-based chemotherapy) of the BETH trial, and arm 1 (3-4 cycles of anthracycline- and cyclophosphamide-based chemotherapy followed by 3-4 cycles of taxane plus trastuzumab plus pertuzumab) of the KAITLIN trial.

Women selected for the primary analysis were aged 45 years and younger with known premenopausal status (with a secondary analysis in women aged 46-55 years) with available archived frozen serum samples taken pretreatment and/or at the end of therapy, and at 36 months (±6 months) from randomization. Thus, there were a minimum of 2 matched samples in all cases allowing analysis of pretreatment and/or end of therapy with postrecovery ovarian function. In both trials, premenopausal status was defined as less than 12 months since last menstrual period prior to study entry. No biochemical evidence of premenopausal status was required.

### Hormone assays

Identified serum samples were centrally measured for AMH, FSH, and E2 using Roche Elecsys assays using a Roche Diagnostics Cobas e801 analyzer. For AMH (Elecsys AMH Plus), the limit of detection (LOD) was 0.01 ng mL^−1^ (0.07 pmol L^−1^) and limit of quantification (LOQ) was 0.03 ng L^−1^ (0.214 pmol L^−1^). In the E2 assay (Elecsys Estradiol III), LOD was 18.4 pmol L^−1^ (5 pg mL^−1^) and LOQ was 91.8 pmol L^−1^ (25 pg mL^−1^). In the FSH assay (Elecsys FSH), LOD was 0.1 IU L^−1^ and LOQ was 1 IU L^−1^. As menstrual function was not recorded, POI was defined using accepted hormonal cutoffs^29^^,^^30^: FSH >25 IU L^−1^ and E2 < 110 pmol L^−1^ at 36 months (primary endpoint) and FSH >40 IU L^−1^ (secondary endpoint).

### Statistical analyses

For diagnosis of POI, a logistic regression model was applied where POI was regressed on AMH measured at the same time, ie, 36 months from randomization. For prediction of POI, a logistic regression model was applied where POI was regressed on AMH measured at baseline or at the end of therapy. AMH values below the LoD were set to 0.07 pmol L^−1^ for all analyses. Further prespecified logistic regression models were calculated to evaluate the effect of AMH when adjusted for additional variables. Due to the expected small numbers of patients and of POI events, regression models with a small number of covariates were used. In particular, the effect of AMH was adjusted by (1) age, (2) FSH, (3) E2, (4) both FSH and E2, and (5) age, FSH, and E2, all with FSH and E2 measured at the corresponding time points with age adjusted at time from randomization. For comparability, the effect of age alone on the presence of POI was estimated by a logistic regression model. For all logistic regression models, the biomarkers (AMH, FSH, E2) were transformed by the natural logarithm.

The performance of a logistic regression model was evaluated by its discriminatory ability and by evaluating the calibration of the model. The discriminatory ability of a logistic regression model was first quantified by the receiver operating characteristic (ROC) curve and the corresponding area under the ROC curve (AUC) with corresponding 95% CI. To assess the calibration of a logistic regression model, evaluating the relationship between the predicted probability and the estimated actual probability, a bootstrap was used to investigate the overoptimism of the intercept and slope of the original model.

For all statistical analyses, the statistical software R (version 4.1.1) was used.

## Results

**Figure 1.**
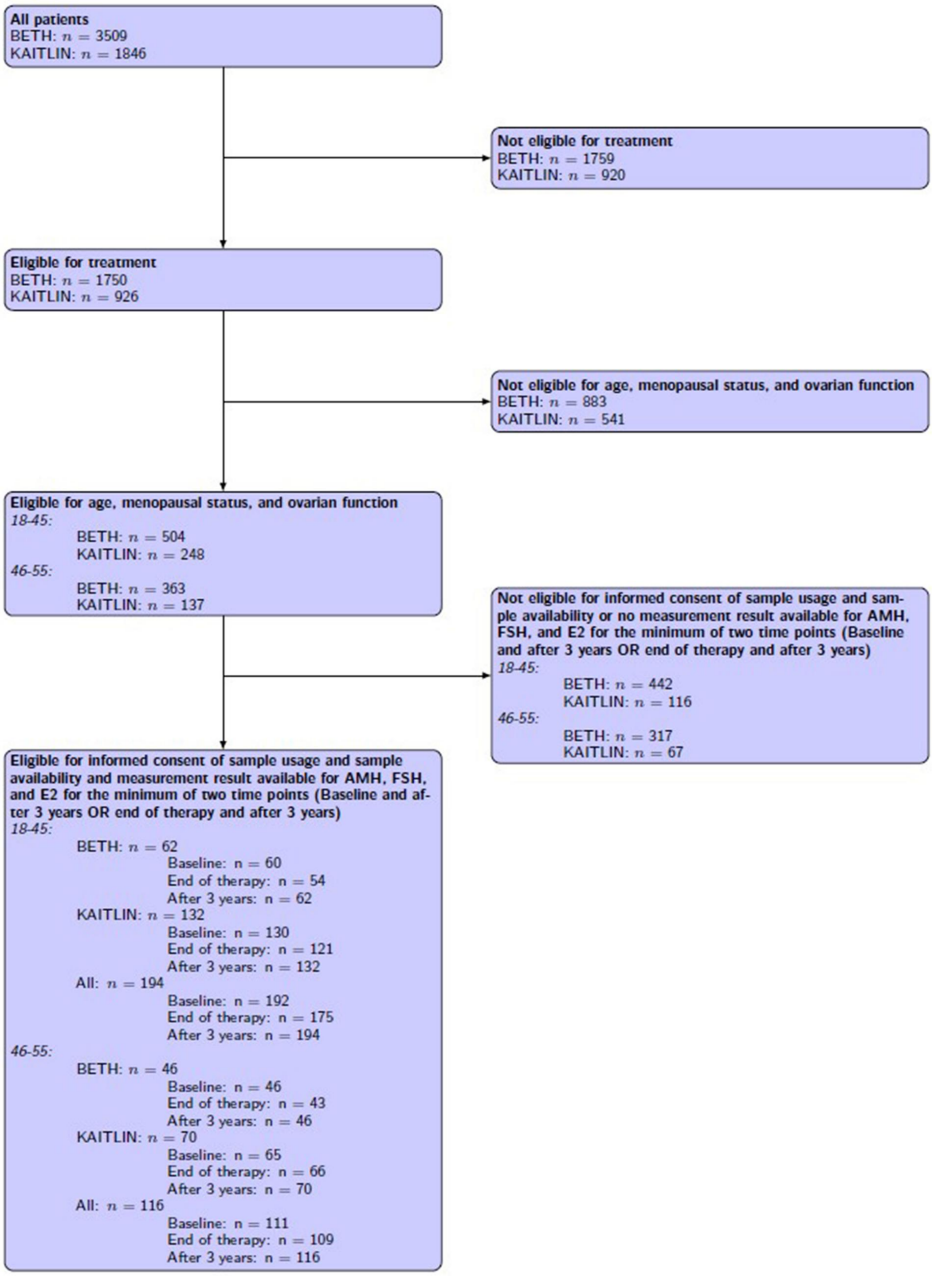
Study flow of participants in the BETH and KAITLIN trials to show selection for the present analysis. Abbreviations: AMH = anti-Müllerian hormone; E2 = estradiol; FSH = follicle stimulating hormone.

A total of 194 patients, 62 from BETH and 132 from KAITLIN, were included in this analysis (Figure 1). Patient characteristics and treatment regimens are detailed in Table 1. Overall, 139 patients (71.6%) received both taxane and anthracycline/cyclophosphamide-based chemotherapy and 55 (28.4%) received a taxane with carboplatin. Pertuzumab was used in addition to trastuzumab in 132 (68.0%) patients.

**Table 1.** Characteristics of patients and treatment regimens.

	All patients	BETH trial	KAITLIN trial
	*n* = 194	*n* = 62	*n* = 132
Age, median (range)	40 (25-45)	41 (27-45)	40 (25-45)
BMI, median (range)	23.4 (17.1-43.9)	23.6 (17.1-38.6)	23.1 (17.7-43.9)
Ethnicity			
White	133 (68.6)	51 (82.3%)	82 (62.1%)
Black	3 (1.5%)	2 (3.2%)	1 (0.8%)
Asian	47 (24.2%)	8 (12.9%)	39 (29.5%)
Other	4 (2.1%)	1 (1.6%)	3 (2.3%)
Unknown	7 (3.6%)	0 (0%)	7 (5.3%)
Type of chemotherapy			
Cyclophosphamide + taxane + anthracycline	82 (42.2%)	0 (0.0%)	82 (62.1)
FU + cyclophosphamide + taxane + anthracycline	57 (29.4%)	7 (11.3%)	50 (37.9%)
Taxane + carboplatin	55 (28.4%)	55 (88.7%)	0 (0%)
Type of taxane			
Docetaxel	146 (75.3%)	62 (100%)	84 (63.6%)
Paclitaxel	46 (23.7%)	0 (0%)	46 (34.8%)
Both	2 (1.0%)	0 (0%)	2 (1.5%)
Type of anti-HER2 therapy			
Trastuzumab alone	62 (32.0%)	62 (100%)	0 (0%)
Trastuzumab + pertuzumab	132 (68.0%)	0 (0%)	132 (100%)
Estrogen receptor status			
Positive	118 (60.8%)	43 (69.4%)	75 (56.8%)
Negative	75 (38.7%)	19 (30.6%)	56 (42.4%)
Unknown	1 (0.5%)	0 (0%)	1 (0.8%)
Progesterone receptor status			
Positive	102 (52.6%)	36 (58.1%)	66 (50.0%)
Negative	90 (46.4%)	26 (41.9%)	64 (48.5%)
Unknown	2 (1.0%)	0 (0%)	2 (1.5%)
Use of GnRHa as adjuvant endocrine therapy			
Yes	30 (15.5%)	4 (6.5%)	26 (19.7%)
No	164 (84.5%)	58 (93.5%)	106 (80.3%)

Abbreviations: BMI = body mass index; FU = fluorouracil; GnRHa = gonadotropin-releasing hormone agonist.

**Figure 2.**
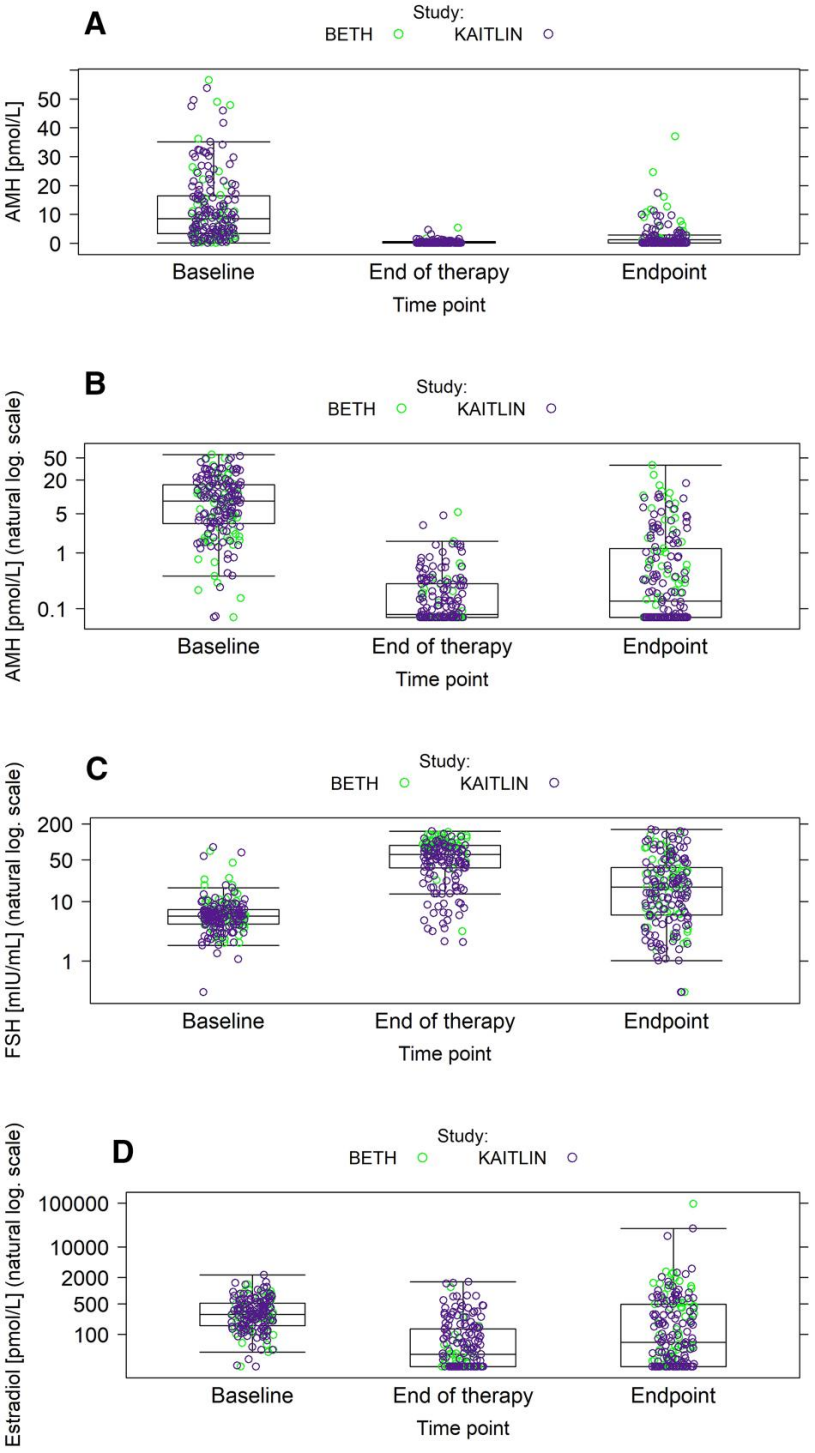
Distribution of AMH, FSH, E2 at 3 time points for patients between 18 and 45 years of age. Distribution of AMH stratified by time points using original scale (A) and natural logarithm scale (B), FSH (C) and E2 (D). Abbreviations: AMH = anti-Müllerian hormone; E2 = estradiol; FSH = follicle stimulating hormone.

During treatment, AMH and E2 concentrations decreased, while FSH increased (Figure 2). There were no differences in AMH, FSH, or E2 between the BETH and KAITLIN cohorts at any of the 3 selected time points; thus, the 2 cohorts were analyzed jointly. AMH declined from pretreatment median 8.44 pmol L^−1^ (IQR 3.36-16.32; *n* = 190) to undetectable levels in 73 out of 175 (41.7%) patients at the end of therapy (median 0.08 pmol L^−1^, IQR 0.07-0.28; *n* = 175), with partial recovery in 138 out of 194 (71.1%) at 36 months (median 0.14 pmol L^−1^, IQR 0.07-1.16; *n* = 194). When using the primary endpoint definition, POI at 36 months was observed in 56 out of 190 women (29.5%) who had AMH available at pretreatment and in 52 out of 175 women (29.7%) who had AMH available at the end of therapy.

**Figure 3.**
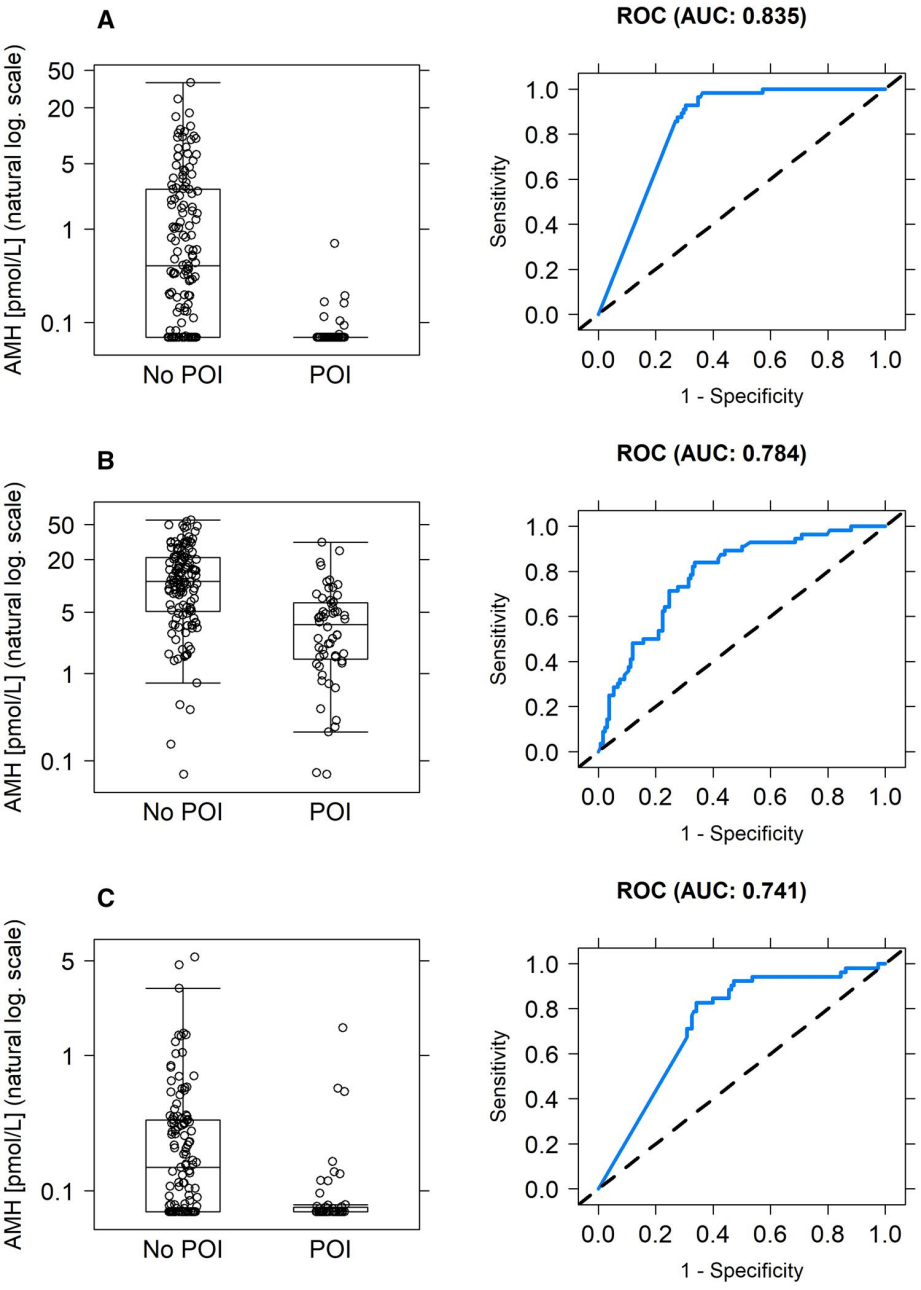
Analysis of diagnostic and predictive value of AMH for premature ovarian insufficiency at different time points. (A) AMH levels at 36 months in women with and without POI, and ROC analysis for diagnostic accuracy at that time point. (B) Predictive value of pretreatment AMH for POI at 36 months, AMH levels at pretreatment in women with and without POI at 36 months, and ROC analysis for predictive accuracy at pretreatment. (C) Predictive value of AMH at the end of treatment for POI at 36 months, AMH levels at the end of treatment in women with and without POI at 36 months, and ROC analysis for predictive accuracy at the end of treatment. Abbreviations: AMH = anti-Müllerian hormone; POI = premature ovarian insufficiency; ROC = receiver operating characteristic.

For diagnosis of POI using AMH values at 36 months from randomization, AMH had an AUC of 0.835 (Table 2; Figure 3), with AUC rising slightly to 0.862 with addition of age, which alone gave a lower AUC of 0.720. Similar results were obtained in the secondary analysis (using an FSH cutoff of 40 IU L^−1^), which reduced the number of women with POI (with pretreatment AMH values available) from 56 to 44 (Table 3). Calibration of the models was also similar for the 2 analyses (Table 3).

**Table 2.** AMH and other biomarkers for diagnosis of premature ovarian insufficiency at 36 months: summary of performance measures for different diagnostic models with internally validated measures using bootstrapping.

Model	Diagnosis (*n* = 194)							
	Primary endpoint (*n* = 56 with POI)				Secondary endpoint (*n* = 44 with POI)			
	AUC	Int.	Slope	LRT	AUC	Int.	Slope	LRT
log. AMH	0.835 (0.835) [0.789 to 0.882]	0.018	0.977	Ref.	0.831 (0.831) [0.787 to 0.874]	0.008	0.980	Ref.
Age	0.720 (0.721) [0.644 to 0.795]	0.062	1.051	—	0.710 (0.711) [0.625 to 0.796]	0.128	1.088	—
log. AMH + Age	0.862 (0.859) [0.812 to 0.912]	0.009	0.944	*P* = .065	0.855 (0.850) [0.804 to 0.907]	−0.014	0.929	*P* = .196

The results of the internal validation are shown in round brackets and the 95% CI for area under the receiver operating characteristic curve (AUC) is shown in square brackets.

Abbreviations: AMH = anti-Müllerian hormone; LRT = likelihood ratio test; POI = premature ovarian insufficiency.

**Table 3.** AMH and other biomarkers at baseline and at the end of therapy for prediction of premature ovarian insufficiency at 36 months: summary of performance measures for different prediction models with internally validated measures using bootstrapping.

Model	Prediction: AMH measured at baseline (*n* = 190)							
	Primary endpoint (*n* = 56 with POI)				Secondary endpoint (*n* = 44 with POI)			
	AUC	Int.	Slope	LRT	AUC	Int.	Slope	LRT
log. AMH	0.784 (0.783) [0.715 to 0.852]	0.018	1.013	Ref.	0.782 (0.783) [0.708 to 0.857]	0.032	1.016	Ref.
Age	0.721 (0.722) [0.646 to 0.797]	0.046	1.042	—	0.707 (0.708) [0.622 to 0.792]	0.114	1.079	—
log. AMH + Age	0.800 (0.795) [0.733 to 0.868]	−0.007	0.978	*P* = .011	0.791 (0.785) [0.716 to 0.867]	−0.009	0.976	*P* = .085
log. AMH + log. FSH	0.784 (0.778) [0.716 to 0.853]	−0.016	0.969	*P* = .163	0.785 (0.779) [0.711 to 0.859]	−0.014	0.972	*P* = .132
log. AMH + log. E2	0.789 (0.783) [0.722 to 0.856]	−0.015	0.971	*P* = .025	0.791 (0.784) [0.718 to 0.864]	−0.013	0.972	*P* = .012
log. AMH + log. FSH + log. E2	0.792 (0.780) [0.726 to 0.858]	−0.037	0.939	*P* = .043	0.797 (0.782) [0.727 to 0.866]	−0.048	0.934	*P* = .022
log. AMH + log. FSH + log. E2 + Age	0.808 (0.793) [0.743 to 0.874]	−0.045	0.919	*P* = .006	0.805 (0.786) [0.732 to 0.877]	−0.068	0.907	*P* = .017

Model	Prediction: AMH measured at the end of therapy (*n* = 175)							
	Primary endpoint (*n* = 52 with POI)				Secondary endpoint (*n* = 40 with POI)			
	AUC	Int.	Slope	LRT	AUC	Int.	Slope	LRT
log. AMH	0.741 (0.742) [0.670 to 0.812]	0.036	1.047	Ref.	0.696 (0.697) [0.615-0.777]	0.150	1.123	Ref.
Age	0.726 (0.728) [0.647 to 0.805]	0.055	1.055	—	0.701 (0.702) [0.610 to 0.790]	0.182	1.132	—
log. AMH + Age	0.785 (0.781) [0.711 to 0.859]	−0.003	0.995	*P* = .015	0.741 (0.736) [0.654 to 0.828]	0.011	1.002	*P* = .039
log. AMH + log. FSH	0.793 (0.790) [0.724 to 0.862]	−0.016	0.963	*P* = .003	0.750 (0.743) [0.671 to 0.829]	−0.028	0.958	*P* = .005
log. AMH + log. E2	0.756 (0.751) [0.682 to 0.830]	−0.012	0.973	*P* = .12	0.717 (0.708) [0.631 to 0.802]	−0.028	0.963	*P* = .238
log. AMH + log. FSH + log. E2	0.794 (0.784) [0.725 to 0.863]	−0.039	0.924	*P* = .011	0.748 (0.731) [0.668 to 0.828]	−0.096	0.890	*P* = .020
log. AMH + log. FSH + log. E2 + Age	0.814 (0.799) [0.747 to 0.882]	−0.058	0.896	*P* = .002	0.772 (0.749)	−0.128	0.856	*P* = .007

The results of the internal validation are shown in round brackets and the 95% CI for area under the receiver operating characteristic curve (AUC) is shown in square brackets.

Abbreviations: AMH = anti-Müllerian hormone; E2 = estradiol; FSH = follicle stimulating hormone; LRT = likelihood ratio test; POI = premature ovarian insufficiency.

Pretreatment AMH concentration was higher in women who did not have POI at 36 months than in those who did, with a median value of 11.4 pmol L^−1^ (IQR 5.1-20.0; *n* = 134) vs 3.6 pmol L^−1^ (IQR 1.5-6.3; *n* = 56). Analysis of pretreatment biomarkers for prediction of POI at 36 months showed that AMH and age were significant predictors of POI, with AUCs of 0.784 and 0.721, respectively (Table 3; Figure 3), which improved to 0.800 in combination. Addition of FSH and E2 to AMH, alone or in combination, had minimal effect on the predictive value, and the combination of all 3 hormones with age slightly improved prediction to 0.808 but with a reduction in calibration (notably in slope) with more variables included (Table 3). Analysis by secondary endpoint showed similar results.

**Figure 4.**
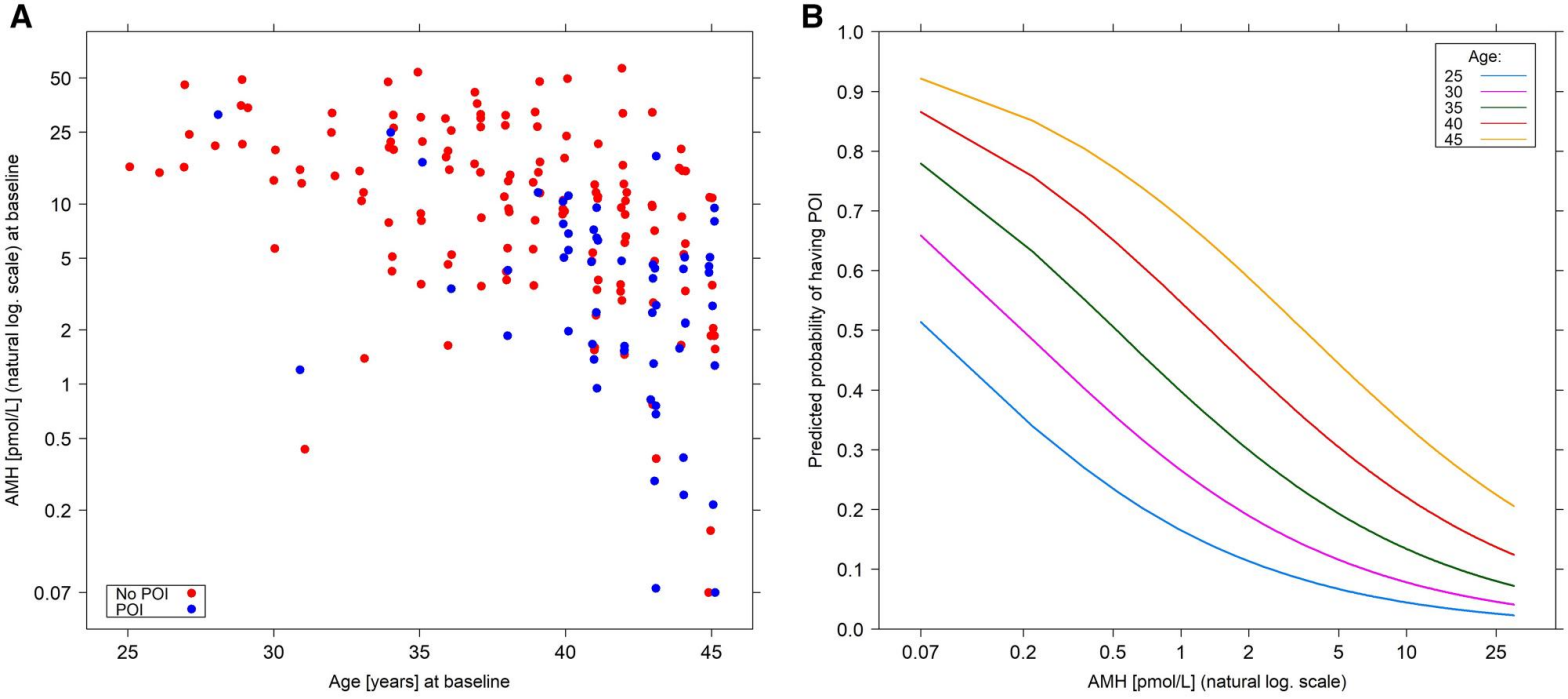
(A) Distribution of baseline AMH and age for development of premature ovarian insufficiency at 36 months. Blue symbols indicate women who developed POI, red symbols indicate women who did not. (B) Results of model for baseline AMH (log scale) against risk of POI at 36 months for a range of exemplar ages. Abbreviations: AMH = anti-Müllerian hormone; POI = premature ovarian insufficiency .

Given this analysis and its potential clinical relevance, baseline AMH and age were selected for model derivation. To aid in clinical application, risk of POI was calculated across a range of AMH and age values. Figure 4 shows the distribution of POI across the full range of AMH values and risk of POI by exemplar 5-year age increments (Figure 4).

Risk of POI at 36 months was also analyzed by biomarker measurement at the end of treatment. Despite the low and relatively narrow range of AMH concentrations at this time point (Figure 2), AMH was again a significant predictor of later POI with AUC of 0.741 (Figure 3; Table 3). Age was less predictive (AUC = 0.726), but addition of age yielded a relatively greater predictive value than analysis of baseline AMH alone, increasing the value of AUC to 0.785. Addition of FSH and E2 to AMH had little value and gave similar AUC to that found with the addition of age (0.794), and the combination of all hormones and age gave AUC of 0.814 (Table 3). Analysis by secondary endpoint showed worse performance for all variables.

Since both baseline and end of treatment AMH levels were predictive of POI at 36 months, their combination was also analyzed. This gave a slightly higher AUC of 0.808 (*n* = 171) than AMH level alone, rising further with addition of age to 0.820.

As gonadotropin-releasing hormone agonist (GnRHa) coadministration may affect FSH, E2 and AMH levels, we performed a sensitivity analysis after excluding the 26 or 30 women (varying between analyses) with a record of any time administration of a GnRHa by month 36. Similar results were observed (Table S1).

A second sensitivity analysis was conducted after excluding the 4 patients who were categorized as premenopausal but were found to have FSH >25 IU L^−1^ at baseline. Consistent results were observed (Table S2).

In women aged 46-55 years (*n* = 116), as expected, AMH levels were reduced with a baseline median value of 0.40 pmol L^−1^ (IQR 0.07-1.27; *n* = 111). At the end of treatment, AMH was undetectable in 101 out of 109 (92.7%) women, with minimal recovery at 36 months. AMH was detectable in only 8 out of 109 (7.3%) women at the end of treatment, and in 5 out of 116 (4.3%) women at 36 months; therefore, further analyses were not undertaken.

## Discussion

Assessment of ovarian function in women with cancer receiving systemic treatment is of growing importance, as also recognized by the American Society for Clinical Oncology.^31^ It is of particular importance in ensuring optimal adjuvant endocrine treatment choices where indicated, specifically regarding the need to add ovarian function suppression to tamoxifen or an aromatase inhibitor, as well as in the oncofertility counseling to more accurately inform the patients about the risk of treatment-induced POI and help them decide whether or not to access fertility preservation strategies before starting chemotherapy. However, despite its relevance, major uncertainties remain in the assessment of posttreatment ovarian function regarding both diagnostic criteria and the potential for late recovery.^32^^,^^33^

In this biomarker analysis of 2 RCTs, we explored the value of AMH as both a diagnostic and a predictive test for posttreatment loss of ovarian function in patients with HER2-positive early breast cancer. We used samples from 2 large RCTs reflecting current chemotherapy and HER2-directed regimens; moreover, we applied 2 cutoffs of FSH currently accepted as the most relevant for diagnosis of loss of ovarian function in this context. We explored AMH alone as well as in combination with age and hormonal variables as a predictor at pretreatment baseline and at the end of chemotherapy, thus at a time point relevant to decisions regarding subsequent endocrine treatment.

Posttreatment samples were taken at 36 months (±6 months) from trial randomization. This time point was selected as ovarian function has typically recovered by then in most women.^11^ Although some patients may show a more delayed apparent recovery, it is unclear whether such late recoveries are sustained or merely represent a short-term period of ovarian activity. AMH showed good diagnostic discrimination for POI, performing similarly with the 2 FSH cutoff values. This is in line with previous analyses using older chemotherapy regimens,^22^^,^^26^^,^^34^ providing evidence for the generalizability of this finding. Although this information was not recorded in the databases of the RCTs, a small number of women may have been administered GnRHa at the 36-month time point. This treatment would be expected to suppress FSH levels, potentially resulting in a woman being wrongly classified as “no POI.” However, the sensitivity analysis excluding patients who received a GnRHa at any time after completing chemotherapy showed very similar diagnostic and predictive accuracy as the main analysis.

The present results provide further evidence to support the predictive value of pretreatment AMH for posttreatment loss of ovarian function, with an AUC of 0.784. Age was less predictive but added to the value of AMH with the combination resulting in an AUC of 0.800. Importantly for clinical utility, addition of FSH and E2 measurement was of minimal added value and reduced calibration accuracy. Thus, the final constructed model used only AMH and age. While the value of AMH in the prediction of posttreatment loss of ovarian function has been shown previously,^19^^,^^21^^,^^25^^,^^35^ confirmation from a larger and more contemporary cohort strengthens evidence for its clinical utility. We were also able to construct a model providing risk of subsequent POI by AMH across a range of ages, providing visual representation of this analysis for use in a clinical context. Notably, these results should be considered in the context of the treatment received by the patients, ie, chemotherapy and HER2-directed regimens. Further validation of these results is needed, particularly in the other breast cancer subtypes and with new treatment regimens. So far, very limited evidence exists on the gonadotoxicity of immunotherapy, CDK4/6 inhibitors, or PARP inhibitors which are currently used together or following chemotherapy for the management of triple-negative and/or high-risk hormone receptor-positive/HER2-negative disease.^36^ Addressing this issue should be considered a research priority in the oncofertility care of young women with breast and other cancers.^31^

AMH measurement at the end of treatment also predicted posttreatment ovarian function loss with AUC of 0.741 but was not as accurate as measurement at baseline. Similar to the baseline analysis, age was less predictive alone but more relevant when assessed in combination with AMH; addition of FSH and E2 were of little value. The combination of AMH measurements at both time points (baseline and posttreatment) increased AUC up to 0.808 (vs 0.784) and, when age was added, it increased to 0.820 (vs 0.800). This predictive accuracy of AMH at the end of treatment is impressive given the low concentrations observed at that time and the narrower range than at baseline. End-of-treatment AMH may provide clinically relevant information in those patients where baseline AMH measurement is not conducted; nonetheless, the combination of both measurements exhibits the strongest value to predict POI.

Among study limitations, it should be considered that this biomarker analysis was not preplanned in the original protocols of the BETH and KAITLIN RCTs. Although the collection of samples was prospectively performed at predefined time points, there was some variation in the timing of sampling. Because the duration of treatment varied by regimen, so did the posttreatment recovery period. Additionally, while we used 2 cutoff values for FSH, there remains no “gold standard” for FSH and E2 values for indicating POI: however, there were only small differences in analytic performance between the 2 cutoff values. The use of an immunoassay for E2 in this study instead of mass spectroscopic methods may have potentially impacted our results, although this might have been more important if a lower E2 cutoff had been used, as the value used is within the performance range of the immunoassay. Since immunoassay methods are more widely available in clinical practice than mass spectroscopic methods, selection of immunoassay in the study adds generalizability.

In conclusion, the present biomarker analysis of the BETH and KAITLIN RCTs supports the value of AMH in the diagnosis and prediction of POI in young women treated for breast cancer using modern chemotherapy regimens and anti-HER2 monoclonal antibodies. We showed that AMH in combination with age exhibits predictive value when measured either prior to or at the end of treatment (albeit the accuracy appears to be reduced at the end of treatment). We present a model with graphical visualization that clinicians and patients may find of value for informing fertility preservation discussions and potentially in aiding shared decision-making regarding the use of ovarian suppression as part of adjuvant endocrine management. Further validation in larger prospective cohorts is required.

## Supplementary Material

djaf198_Supplementary_Data

## Data Availability

Individual participant data that underlies the results reported in this article, after de-identification (text, tables, figures, and appendix) can be made available for further sharing upon reasonable request. Requests concerning the data supporting the findings of this study should be directed to rotkreuz.datasharingrequests@roche.com for consideration.
