# Supplementary material for: Predicting ovarian function loss after chemotherapy and anti-HER2 therapy in young breast cancer patients
Source: J Natl Cancer Inst. 2025 Aug 12;117(11):2317–26. doi: 10.1093/jnci/djaf198 (PMC12597502; doi:10.1093/jnci/djaf198)
Supplement: djaf198_Supplementary_Data [file djaf198_supplementary_data.pdf]

Supplementary Material

Table S1. Sensitivity analysis excluding patients who received GnRHa therapy – AMH and age for diagnosis, AMH at baseline, and AMH at end of therapy for prediction of premature ovarian insufficiency at 36 months: performance measures for different prediction models with internally validated measures using bootstrapping.

		***Diagnosis** (n = 164)*						
	**Primary Endpoint (n = 53 with POI)**				**Secondary Endpoint (n = 42 with POI)**			
	AUC	Int.	Slope	LRT	AUC	Int.	Slope	LRT
log. AMH	0.842 (0.843) [0.792 – 0.893]	0.016	0.990	Ref.	0.837 (0.837) [0.789 – 0.884]	0.022	0.981	Ref.
Age	0.708 (0.709) [0.626 – 0.790]	0.048	1.058	-	0.703 (0.703) [0.612 – 0.794]	0.125	1.106	-
log. AMH + Age	0.863 (0.857) [0.809 – 0.917]	0.015	0.946	p = 0.286	0.857 (0.847) [0.802 – 0.912]	0.005	0.916	p = 0.502
		***Prediction: AMH measured at baseline** (n = 160)*						
	**Primary Endpoint (n = 53 with POI)**				**Secondary Endpoint (n = 42 with POI)**			
	AUC	Int.	Slope		AUC	Int.	Slope	
log. AMH	0.777 (0.779) [0.703 – 0.851]	0.045	1.039	Ref.	0.781 (0.785) [0.704 – 0.859]	0.064	1.041	Ref.
Age	0.709 (0.710) [0.627 – 0.791]	0.060	1.071	-	0.698 (0.699) [0.607 – 0.789]	0.156	1.140	-
log. AMH + Age	0.792 (0.787) [0.719 – 0.865]	0.008	0.987	p = 0.022	0.789 (0.784) [0.710 – 0.867]	0.011	0.985	p = 0.12
		***Prediction: AMH measured at end of therapy** (n = 149)*						
	**Primary Endpoint (n = 49 with POI)**				**Secondary Endpoint (n = 38 with POI)**			
	AUC	Int.	Slope		AUC	Int.	Slope	
log. AMH	0.729 (0.730) [0.654 – 0.803]	0.043	1.053	Ref.	0.691 (0.691) [0.608 – 0.773]	0.147	1.129	Ref.
Age	0.713 (0.713) [0.628 – 0.799]	0.058	1.060	-	0.690 (0.691) [0.595 – 0.786]	0.173	1.145	-
log. AMH + Age	0.782 (0.777) [0.705 – 0.860]	0.005	0.989	p = 0.031	0.740 (0.731) [0.650 – 0.830]	0.004	0.987	p = 0.071

The results of the internal validation are shown in round brackets and the 95% confidence interval for Area under the Receiver Operating Characteristic Curve (AUC) is shown in square brackets.

Abbreviations: POI, premature ovarian insufficiency; AMH, anti-Müllerian hormone; FSH, follicle stimulating hormone; E2, estradiol

Table S2. Sensitivity analysis excluding patients who fulfill biochemical definition of ovarian loss at baseline – AMH and age for diagnosis, AMH at baseline, and AMH at end of therapy for prediction of premature ovarian insufficiency at 36 months: performance measures for different prediction models with internally validated measures using bootstrapping.

	***Diagnosis** (n = 190)*							
	**Primary Endpoint (n = 53 with POI)**				**Secondary Endpoint (n = 41 with POI)**			
	AUC	Int.	Slope	LRT	AUC	Int.	Slope	LRT
log. AMH	0.836 (0.835) [0.789 – 0.884]	0.006	0.973	Ref.	0.832 (0.831) [0.787 – 0.876]	-0.006	0.970	Ref.
Age	0.713 (0.714) [0.635 – 0.791]	0.048	1.048	-	0.699 (0.701) [0.610 – 0.789]	0.139	1.097	-
log. AMH + Age	0.862 (0.858~~2~~) [0.812 – 0.913]	-0.003	0.938	p = 0.078	0.854 (0.845) [0.801 – 0.907]	-0.031	0.915	0.264
	***Prediction: AMH measured at baseline** (n = 186)*							
	**Primary Endpoint (n = 53 with POI)**				**Secondary Endpoint (n = 41 with POI)**			
	AUC	Int.	Slope	LRT	AUC	Int.	Slope	LRT
log. AMH	0.778 (0.776) [0.707 – 0.848]	0.015	1.009	Ref.	0.772 (0.771) [0.695 – 0.850]	0.031	1.013	Ref.
Age	0.715 (0.716) [0.637 – 0.792]	0.045	1.048	-	0.696 (0.697) [0.607 – 0.784]	0.135	1.098	-
log. AMH + Age	0.795 (0.789) [0.726 – 0.865]	-0.013	0.973	p = 0.012	0.783 (0.776) [0.705 – 0.861]	-0.017	0.969	p = 0.089
	***Prediction: AMH measured at end of therapy** (n = 171)*							
	**Primary Endpoint (n = 49 with POI)**				**Secondary Endpoint (n = 37 with POI)**			
	AUC	Int.	Slope	LRT	AUC	Int.	Slope	LRT
log. AMH	0.738 (0.737) [0.664 – 0.811]	0.041	1.040	Ref.	0.688 (0.688) [0.603 – 0.773]	0.141	1.107	Ref.
Age	0.718 (0.718) [0.636 – 0.799]	0.052	1.047	-	0.685 (0.686) [0.591 – 0.779]	0.171	1.121	-
log. AMH + Age	0.780 (0.776) [0.704 – 0.857]	0.006	0.994	p = 0.022	0.730 (0.723) [0.638 – 0.821]	0.017	0.998	p = 0.07

The results of the internal validation are shown in round brackets and the 95% confidence interval for Area under the Receiver Operating Characteristic Curve (AUC) is shown in square brackets.

Abbreviations: POI, premature ovarian insufficiency; AMH, anti-Müllerian hormone; FSH, follicle stimulating hormone; E2, estradiol; LRT, likelihood ratio test
